# Unraveling Calcium Absorption and Distribution in Peach and Nectarine during Fruit Development through ^44^Ca Isotope Labeling

**DOI:** 10.3390/plants13162287

**Published:** 2024-08-17

**Authors:** Francisca Carrasco-Cuello, Gregory Van der Heijden, Josep Rufat, Estanis Torres

**Affiliations:** 1 Fruticulture Program, IRTA Fruitcentre, Parc AgroBiotech, 25003 Lleida, Spain; 2 Efficient Use of Water in Agriculture Program, IRTA Fruitcentre, Parc AgroBiotech, 25003 Lleida, Spain; 3 INRA de Nancy, Rue d’Amance, 54280 Champenoux, France

**Keywords:** stable isotope, sprays, foliar application, *Prunus persica*, fertilization

## Abstract

Calcium foliar applications are known to effectively enhance peach quality; however, the optimal implementation strategy regarding fruit developmental stages and cultivars remains unclear. In this study, three different moments of fruit Ca applications in peach and nectarine are tested: Early season, Mid-season, and Late season. For this aim, the ^44^Ca isotope was used as a tracer, enabling the quantification and location of the Ca derived from the foliar fertilizer. Stone, flesh, and skin ^44^Ca enrichment was separately analyzed at harvest. The results indicate that Ca absorption in the fruits from external CaCl_2_ applications was influenced by the timing of the application during fruit development, with Late-season applications proving to be the most effective in increasing the Ca content in the fruit, corresponding with a higher fruit size at the application moment. Nevertheless, no differences in the absorption efficiency were found between the three timings of the application. Furthermore, the Ca from the foliar fertilizer in the fruit predominately remained in the flesh, followed by the skin. The Ca derived from the foliar fertilizer reached the stone in all of the experimental situations, but the Early- and Mid-season applications resulted in the highest amount of Ca derived from the fertilizer in this part of the fruit. Interestingly, the peach exhibited a higher Ca absorption efficiency compared to the nectarine, likely due to the presence of trichomes that retain the foliar fertilizer on the fruit surface. In conclusion, the Ca absorption and distribution in peaches depends on the cultivar and timing of the Ca application.

## 1. Introduction

Peach (*Prunus persica* L. Batsch) is a valuable temperate fruit crop which involves different types of fruits (peaches and nectarines) in various shapes (round and flat). Peaches and nectarines are known for being a rich source of bioactive compounds, including flavonoids, anthocyanins, carotenoids, phenolic compounds, and vitamins, and they possess high antioxidant activity. Therefore, consumers highly value quality peaches for their nutritional content and agreeable sensory attributes [1,2]. Its consumption is usually fresh fruit, being a highly perishable one. After harvest, peaches and nectarines experience some physiological and biochemical changes associated with ripening that affect their texture, flavor, and overall fruit quality [3]. Their storage potential and postharvest life depend on various factors, including proper preharvest practices that are essential to maintaining the quality and extending the shelf-life. The use of fertilizers, such as calcium (Ca), and its efficiency play a major role within these practices.

The effect of Ca on enhancing postharvest fruit quality is well known. Ca plays a key role in the structure and stability of both cell walls and cell membranes [4]. Hence, optimal fruit Ca levels have been linked with enhanced fruit firmness, reduced bruising during packing, grading, and transportation, as well as an improved shelf-life [5,6]. Balanced Ca in peaches and nectarines may also mitigate other physiological disorders such as skin cracking [7], corky spot or chilling injury, among others [8,9,10], as well as reduce infections by fungal pathogens such as *Monilinia* spp. [11].

In Spain, most of the soils used for stone fruit production have adequate levels of Ca for vegetative development. Nevertheless, fruit disorders related to Ca deficiency occur due to the limited mobility of Ca within the plant. Ca moves through the transpiration stream via the xylem from the soil to the different parts of the plant, but it is phloem-immobile [12]. Hence, early fruit development is the most critical period for Ca accumulation in most fleshy fruits [13,14]. In the early season, for most fleshy fruits, phloem and xylem sap influxes are similar. However, in the late season, the total sap inflow is principally dominated by the phloem, with xylem sap inflow being insignificant due to the loss of xylem functionality during fruit growth and development [13,15]. This causes a reduction in the Ca flow to most fruits, but does not affect the K, Mg and N flow, which can compromise the postharvest quality of the fruits [16]. Thus, from this point forward, only foliar Ca applications that directly impact the fruit will be able to significantly increase the fruit Ca.

Foliar Ca applications may be especially advantageous for improving the Ca content in fruits if conditions for soil uptake are impaired or if there is excessive early season shoot growth. The authors of [17] reported that the preharvest spray application of CaCl_2_ in peaches substantially reduced the incidence of chilling injury and fruit softening during storage, and delayed climacteric ripening, seemingly by lowering the rates of ethylene production. However, to be effective, foliar Ca applications need to be applied several times during the phenological stages of fruit development [6,18], and the process of fruit Ca absorption from sprays is not yet well understood [5,17]. Several factors can influence the effectiveness of foliar Ca applications. The cuticle (i.e., skin) is the first barrier against the absorption of foliar-applied nutrients [19]. Variations in the fruit type and growth stage lead to differences in the morphologies of the exocarp and cuticle, potentially affecting Ca absorption and utilization by the fruit.

A methodology to study Ca absorption and distribution in fruits where Ca foliar applications have been proposed is the use of Ca isotopes as tracers. Recently, the ^44^Ca isotope was used in apple [20] and sweet cherry [21], and the ^45^Ca isotope was used in oranges [22], to study fruit and foliar Ca absorption. These findings highlight the importance of researching the Ca external supply at different fruit development stages and fruit typologies in relation to the characteristics of their cuticles. The main objective of this assay was to unravel the differences between peaches and nectarines in Ca absorption in different developmental stages using ^44^Ca as a tracer. As far as we are concerned, no study utilizing Ca isotopes as tracers has been conducted in peaches and nectarines. Consequently, we developed fruit-targeted ^44^Ca-labeled applications for two cultivars (nectarine and peach) at three different fruit developmental stages (Early, Mid, and Late).

## 2. Results

### 2.1. Total Ca and Ca Derived from the Foliar Fertilizer

**Figure 1:**
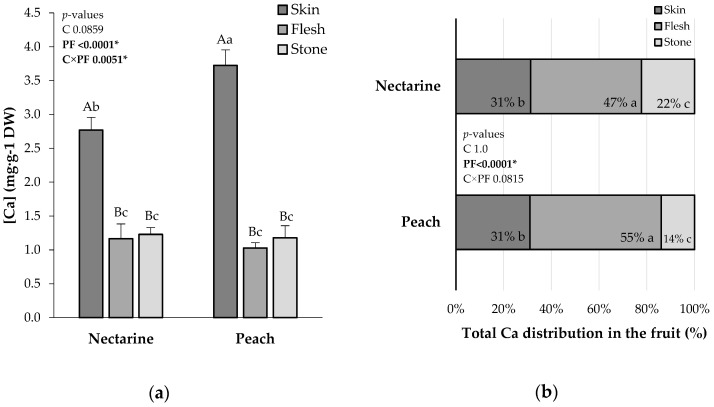
(**a**) Ca concentration ([Ca], mg·g^−1^ DW) per part of the fruit (PF) (skin, flesh, and stone) in the cultivars (C) ‘*Esmeralda*’ (nectarine) and ‘*Tardibelle*’ (peach) at harvest date for untreated samples. (**b**) Distribution at harvest of total Ca (Ca distribution in the fruit, % DW) per parts of the fruit (PF) (skin, flesh, and stone) in the cultivars ‘*Tardibelle*’ (peach) and ‘*Esmeralda*’ (nectarine) for untreated samples. Different capital letters indicate statistical differences between the PF inside the C. Different lowercase letters indicate the differences between each PF in both C. The *p*-values are indicated in the top left of the graphs. * Indicates statistical significance.

The total Ca concentration (mg·g^−1^ DW) in the skin, flesh, and stone followed a similar pattern between the peach and the nectarine for untreated fruits. The nectarine cultivar ‘*Esmeralda*’ presented 2.77 mg·g^−1^ DW of Ca in the skin, 1.16 mg·g^−1^ DW in the flesh, and 1.23 mg·g^−1^ DW in the stone. The peach cultivar ‘*Tardibelle*’ presented 3.72 mg·g^−1^ DW in the skin, 1.02 mg·g^−1^ DW in the flesh, and 1.18 mg·g^−1^ DW in the stone. The skin in both cultivars is the most Ca-enriched tissue, but significantly higher in the peach than in the nectarine. Notably, the flesh and the stone presented a similar Ca concentration for both cultivars (Figure 1a).

In terms of the distribution (%) of the total Ca content, ‘*Tardibelle*’ and ‘*Esmeralda*’ followed a similar pattern. The flesh was the most-reached tissue in Ca in both cultivars. The ‘*Esmeralda*’ nectarine presented 31% of the total Ca in the skin, 47% in the flesh, and 22% in the stone. The ‘*Tardibelle*’ peach represented 31% of the total Ca in the skin, 55% in the flesh, and 14% in the stone. When comparing the fruit parts, the flesh was the one with the highest Ca content in each cultivar (Figure 1b).

**Figure 2:**
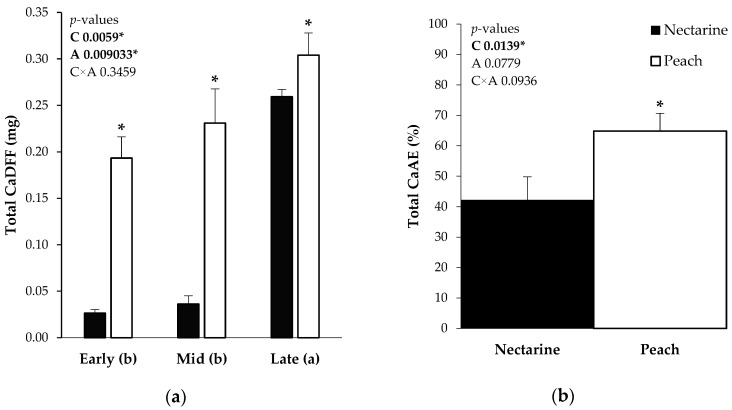
(**a**) Total Ca derived from the foliar fertilizer (Total CaDFF (mg)) in the fruit for nectarine (black) and peach (white) in each Ca application (Early, Mid, and Late) represented in the abscissa axis. (**b**) Total Ca absorption efficiency (CaAE (%)) in nectarine (black) and peach (white). Total CaAE (%) was calculated as the relationship between the total Ca derived from the fertilizer inside of the fruit (CaDFF) and the Ca absorbed per fruit. The *p*-values are indicated in the top left of the graphs. * Indicates statistical differences between the cultivars. Different letters indicate the differences between the applications in the abscissa axis. C (cultivar): nectarine and peach, A (Ca application): Ealy, Mid, and Late.

The absorption of Ca through an external CaCl_2_ application in the fruit was quantified as the total content of the isotope from the fertilizer and was defined as the Ca derived from the fertilizer (CaDFF). This parameter represents the Ca content that was absorbed by the CaCl_2_ application. The total CaDFF was significantly higher in the peach cultivar than in the nectarine. In general, the fruits treated Early (0.10 mg) and Mid-season (0.12 mg) presented significantly less CaDFF than the Late-season Ca application without the interaction with the cultivar (Figure 2a).

It is worth mentioning that, when the Late-season Ca application was performed, the fruit fertilizer absorbed was higher due to the higher fruit size. Consequently, to compare the applications, the Ca absorption efficiency (CaAE) was calculated.

### 2.2. Ca Absorption Efficiency and Ca Fruit Distribution from ^44^CaCl_2_ Application

The CaAE is defined as the relationship between the CaDFF and the ^44^Ca-labeled applications in the fertilizer absorbed by each fruit. There were no significant differences between the Early-, Mid-, and Late-season Ca applications in the CaAE, which suggests that the fruit capacity to absorb Ca was independent from the application moment. Nevertheless, there were significant differences between the cultivars. The peach ‘*Tardibelle*’ presented a significantly higher total CaAE (63.40%) than the nectarine ‘*Esmeralda*’ (41.31%) (Figure 2b).

**Figure 3:**
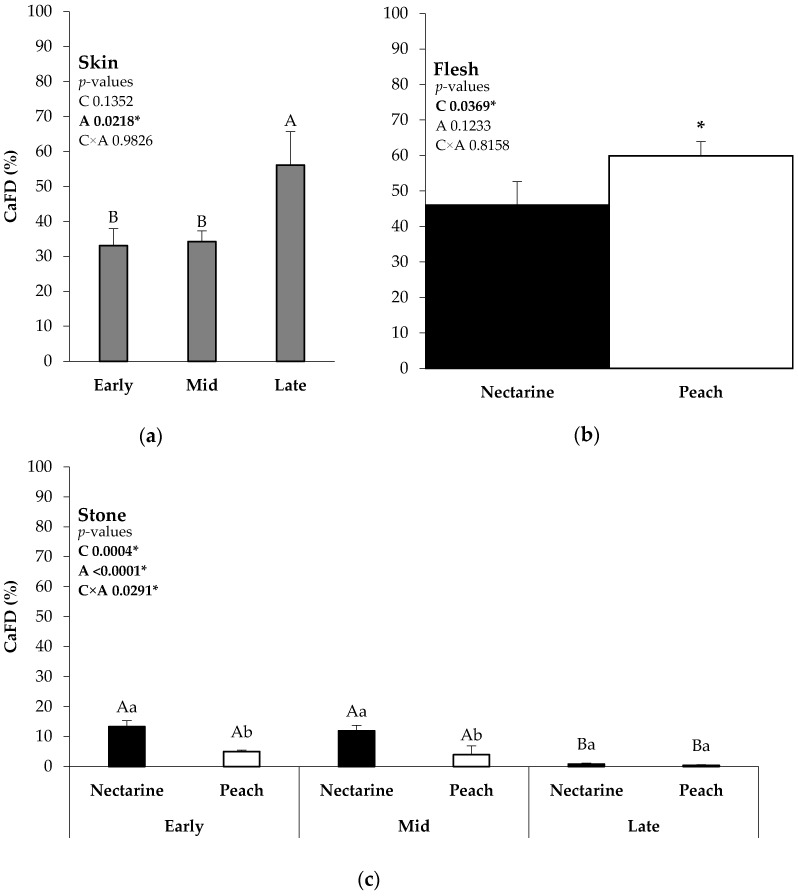
Ca fruit distribution rate (CaFD %) calculated as the CaDFF found in each part of the fruit (skin (**a**), flesh (**b**), and stone (**c**)) related to the total CaDFF found in the fruit. The *p*-values are indicated in the graphs. * Indicates statistical significance. Different capital letters indicate the differences between the Ca applications in both cultivars. Within the same Ca application moment, different lowercase letters indicate significant differences between cultivars. C (cultivar): nectarine and peach, A (Ca application): Ealy, Mid, and Late.

The Ca fruit distribution rate (CaFD (%)) represents the percentage of the total CaDFF found in the fruit distributed per part of the fruit. The Late-season Ca application significantly increased the CaFD in the skin compared to the Early- and Mid-season Ca applications (Figure 3a).

In the flesh, the peach ‘*Tardibelle*’ presented a higher CaFD than the nectarine ‘*Esmeralda*’. There were no differences between applications in this part of the fruit regarding the CaFD (Figure 3b).

The nectarine ‘*Esmeralda*’ presented the highest CaFD in the stone compared to the peach ‘*Tardibelle*’ in the Early- and Mid-season Ca applications. Conversely, in the Late-season Ca application, there were no differences between the nectarine and the peach. On the other hand, the Early- and Mid-season Ca applications presented a significantly higher amount of CaFD in the stone compared to the Late-season Ca application. Furthermore, the stone was the least part of the fruit enriched with the isotope in every situation compared to the skin and the flesh, as was expected (Figure 3c).

## 3. Discussion

Comparing the total Ca among the three fruit parts analyzed, the skin exhibits a significantly higher total Ca concentration in both cultivars. In contrast, the flesh presents the highest Ca content, as it contributes the most to the total fruit weight. Ca accumulation in the fruit correlates with cumulative transpiration, as Ca movement is primarily associated with water flow [12,23]. Ca uptake inside of the fruit from the pedicel is influenced by the water flow and the fruit growth [24,25]. Within the fruit, water flows from the pedicel to the skin, where water loss occurs through fruit transpiration [26], which can be related to the significantly higher accumulation of Ca in the skin in terms of the concentration compared to the flesh and the stone.

The Late-season Ca application resulted in the highest total CaDFF, but not CaAE, likely due to the increment in the fruit size. Ref. [21] observed that the Ca applications in the early stages were the most efficient in sweet cherry, probably correlated with the highest fruit transpiration and cell division stage. In peaches, the period of higher fruit transpiration and apparent functionality of the lenticels was also the early stage [27]. Nevertheless, peach fruit cracks tend to increase with the fruit development process, especially during the last developmental period, due to the rapid fruit water accumulation [28].

The Early- and Mid-season Ca applications exhibited the highest CaFD, leading to higher Ca levels in the stone. The penetration of Ca from the skin to the stone appears to be related to a diffusion process, with the time between the Ca application and the harvest date determining the Ca penetration from the treated tissue (the skin) to other parts of the fruit. Additionally, the fruit size increment (as the distance between the skin and the stone) may contribute to promoting the stone Ca location in the Early- and Mid-season applications. Unexpectedly, the CaDFF was found in the stone even in Late-season application, but more research is needed to understand if it remains in the stone surface or if the CaDFF penetrates the stone. Previous studies have demonstrated that conducting the Ca application closest to the harvest date yields the most effective mitigation of fruit Ca-related disorders in apples [18]. This could be related to the higher amount of Ca that impacts the fruit when the fruit size increases and to the Ca location in the skin.

Interestingly, foliar spray applications of CaCl_2_ directly impact on the skin, with Ca penetration occurring against the fruit water flow. Nonetheless, the results presented herein showed that Ca penetrates from the skin to the stone in both cultivars and applications. This phenomenon may be attributed to a passive mechanism, whereby Ca diffusion follows the concentration gradient, influencing the solute entrance across the fruit cuticle cracks or fruit lenticels [22], and movement from the most concentrated tissue (the treated skin) to the less Ca-concentrated ones (the flesh and the stone). Further studies are required to elucidate the pathways of Ca absorption in fruits and vegetables following external foliar applications, as well as its distribution mechanism within the different parts of the fruit.

The use of CaCl_2_ is one of the most recommended foliar Ca applications in agriculture, possibly due to its effectiveness in having a point of deliquescence lower than other Ca salts [29]. It has been employed in Ca foliar nutrition studies in various crops, including peaches, tomato, apple, orange, and cherry, among others [9,18,21,22,30].

Based on the findings presented herein, the peach showed a higher CaAE compared to the nectarine when treated with CaCl_2_ foliar fertilizer, although no significant differences were observed between applications. This suggests that peaches absorb more Ca into their tissues through foliar Ca applications than the nectarine, which could be due to the differences in the skin morphology between the cultivars.

The peach and nectarine skins act as a hydrophobic barrier against water [31]. The main difference between peaches and nectarines is the presence of trichomes in peaches. Trichomes, considered a non-polar surface covered by alkane chains with a low wax proportion, may contribute to maintaining the foliar fertilizer in the fruit surface [32]. In contrast, nectarines lack trichomes, and their skin only presents the cuticle as a hydrophobic barrier. In other words, the absence of trichomes in nectarines appears to result in the foliar fertilizer slipping down the fruit faster, enhancing the foliar fertilizer reaching the spray run-off point sooner.

In contrast, peach trichomes appear to retain droplets, thereby increasing the efficiency of foliar fertilizer by prolonging the exposure time. However, these phenomena can trigger peach skin streaking, a form of discoloration related to water droplets formed in the fruit surface [33], which should be considered when treating peach fruit. These results align with [32], who reported that the presence of trichomes improved the water sorption capacity compared to isolated cuticles with trichomes mechanically removed. Surprisingly, the CaAE from CaCl_2_ in nectarine fruit (41%) was similar to the Ca absorption efficiency reported before in apple by [20], possibly due to the smooth skin in both fruits.

In summary, peach Ca foliar fertilization should be defined for both fruit typologies and their developmental stages to optimize the technique. Late-season Ca applications in both peaches and nectarines prove to be the most effective application for increasing the Ca content in the perishable parts of the fruit, but not the CaAE. Conversely, Early- and Mid-season Ca applications are preferable for increasing Ca in the stone. Peaches exhibit superior foliar fertilizer absorption compared to nectarines, possibly due to differences in the presence or absence of trichomes and waxes in the epidermis. These findings underscore the necessity for further research in the foliar nutrition of Ca in peach crops.

## 4. Materials and Methods

### 4.1. Experimental Conditions and Plant Material

Eight *Prunus persica* trees, four per each of two late-ripening cultivars (‘*Esmeralda*’ and ‘*Tardibelle*’) with a uniform size and productivity, were selected from a commercial orchard located in Aitona, Spain (41°31′15″ N 0°26′37″ E, 130.95 m), on a Calcaric Regosols/Haplic Calcisols according to the World Reference Base for Soil Resources. Trees were selected based on the uniform flower clusters and growth. ‘*Esmeralda*’ trees were 7 years old, grafted on ‘*Garnem*’ rootstock, and ‘*Tardibelle*’ trees were 10 years old, grafted on ‘*GF-677*’ rootstock, both trained to an open vase system and spaced at 5 × 3 m. ‘*Esmeralda*’ is a yellow-fleshed nectarine cultivar and ‘*Tardibelle*’ is a yellow-fleshed peach cultivar, and the main differences between them is the presence of skin trichomes in the peach cultivar and the smooth skin of the nectarines. Both cultivars presented the pit-hardening point 30 days before the first application. The orchard was managed using traditional practices. The experiment was conducted from May to September 2023, and nine fruits per tree were selected based on their uniform size and similar position in the canopy. Each Ca application was performed on 3 fruits per tree, resulting in a total of 72 fruits in the assay (3 fruits × 3 applications × 4 trees × 2 cultivars) (Table 1).

### 4.2. ^44^CaCl_2_ Preparation and Applications

The nutrient solution (foliar fertilizer) was made by mili-Q water with a final 500 mg·kg^−1^ calcium chloride solution made of 97 atom% ^44^CaCl_2_. The ^44^CaCl_2_ was prepared by dissolving ^44^CaCO_3_ (enriched to 97%, Cambridge Isotope Laboratories, Inc., USA) in concentrated HCl, based on [20]. No surfactant was used in the assay. Three different ^44^Ca applications were implemented based on the fruit developmental stages: Early season (88 and 94 DAFB), Mid-season (119 and 124 DAFB), and Late season (161 and 166 DAFB), corresponding each application with one group of 3 fruits per tree. This implies that each tree contained three fruit groups, each subjected to a different Ca application (Table 1).

Fruit isotopic fertilizer was applied early in the morning on sunny days. A defined volume of the foliar fertilizer was applied with a small brush directly to the fruit, collecting the excess. The same volume of the foliar fertilizer was applied at every timing and cultivar, and the volume absorbed by each fruit was measured by subtracting the measured unabsorbed volume from the applied volume for each fruit.

### 4.3. Collection and Processing of Samples

From each Ca application group, one shoot per tree was selected based on the uniform length and position in the canopy. This selection process aimed to account for any potential disadvantage caused by the natural abscission of the fruit during the assay. Hence, four samples were collected for each replicate, with each sample representing three fruits. The same procedure was performed for the untreated samples. All of the samples were collected at harvest dates, while the weight and size were used for the processing and analysis (Table 1).

Next, the samples were rinsed with deionized water and separated into the following three different parts: the skin (exocarp), flesh (mesocarp), and stone (endocarp and the kernel). The samples were oven-dried, and the dry material was ground and homogenized using a mortar and pestle until powder. The stone powder was initially obtained through manual fragmentation using pruning shears, followed by further processing with a mechanical blender. The DW was measured afterwards.

### 4.4. Ca and ^44^Ca-Labeled Analysis

The Ca content and isotope analysis were measured at the analytical mass spectrometry at INRA of Nancy (France). Mineral content determination was achieved by nitric digestion and the subsequent dilution of the digestion with mili-Q water. The Ca concentration in the skin, flesh, and stone ([Ca], mg·g^−1^ DW) was measured with an atomic emission spectrometer with an 820 MS Analytical Jena inductively coupled mass spectrometer (ICP-MS) (Analytik Jena AG, Jena, Germany) for every part of the fruit. The Ca distribution in the fruit (%) was calculated by the amount of Ca (mg) in the skin, flesh, and stone compared with the total amount of Ca in the fruit (mg).

The ^44^Ca/^40^Ca ratio was measured in a mass spectrometer with a multiple collector and inductively coupled plasma source (MC-ICP MS, Thermo Finnigan Neptune, INRA), following the described methodology by [34,35], and validated by instrument intercalibration [34].

To calculate the Ca derived from the fertilizer (CaDFF), the following formula was applied in every part of the fruit (*i*):

$$CaDFF\left(mg\right)=\frac{\left(\alpha i-\alpha control\right)}{\left(\alpha tracer-\alpha control\right)}\times DW\times [Ca]i$$

CaDFF is defined as the ^44^Ca (mg) content in the fruit that comes from the foliar fertilizer [36]; α_i_ is the Ca isotopic composition of the skin, flesh, or stone that was isotopically labeled (^44^Ca atom%); α_control_ is the ^44^Ca isotopic composition from the natural abundance (^44^Ca atom%); α_tracer_ is the ^44^Ca isotopic composition of the tracing solution (97 atom% ^44^Ca); [Ca]_i_ is the Ca concentration in skin, flesh, or stone (mg·g^−1^ DW); DW is the dry weight either of the skin, flesh, or stone (g). Total CaDFF corresponds with the summatory of the CaDFF in the three parts (skin, flesh, and stone) of the same fruit.

The Ca absorption efficiency (CaAE %) was defined as the relationship between the CaDFF (mg) and the Ca absorbed per fruit, and was calculated by the following formula [37]:

$$CaAE\;\left(\%\right)=\frac{CaDFF\left(mg\right)\times 100}{Ca\;absorbed\;per\;fruit\;(mg)}$$

The Ca absorbed per fruit (mg) was measured by the concentration of ^44^Ca in the foliar fertilizer multiplied by the volume of fertilizer absorbed in each fruit.

The relationship between the CaDFF per part of the fruit and the Total CaDFF was defined as the Ca fruit distribution rate (CaFD %) and represents the percentage of the CaDFF in each part of the fruit compared to the total CaDFF in the fruit, which was calculated with the following formula:

$$CaFD\left(\%\right)=\frac{CaDFF\left(mg\right)\times 100}{Total\;CaDFF\;(mg)}$$

### 4.5. Statistical Analysis

The experiment followed a completely randomized design, comprising 6 treatments (3 Ca applications × 2 cultivars) and 4 replicates each. Data analysis was conducted using an analysis of variance (ANOVA) with JMP Pro-Software (version 16.0, SAS Institute Inc., Cary, NC, USA). An ANOVA was employed to test the main effects of the cultivars (C) (peach and nectarine), Ca applications (A) (Early, Mid, and Late), and parts of the fruit (P) (skin, flesh, and stone). Statistical significance was assumed at *p*-levels < 0.05. Tukey’s test was used to separate the means when required. Parameters are presented in the figures as means ± standard error (SE).

## 5. Conclusions

The following conclusions can be drawn based on our results and the discussion presented: (1) The skin exhibited the highest concentration of Ca in both peach and nectarine, while the Ca content was primarily found in the flesh, followed by the skin and the stone. (2) Peach trichomes enhanced the Ca absorption efficiency of the fertilizer compared to the nectarine. (3) Late-season Ca applications significantly increased the amount of Ca from the foliar fertilizer in the fruit compared to Early- and Mid-season applications, but no significant differences were observed between the applications for the Ca absorption efficiency. (4) Early- and Mid-season applications increased the Ca fruit distribution in the stone more than the Late-season application, which increased the Ca fruit distribution in the perishable part of the fruit.

## Figures and Tables

**Table 1:** Cultivar, harvest date, calcium application, application date, phenological stage (BBCH), and fruit diameter (mm) for Early, Mid, and Late applications. DAFB (days after full bloom); BBCH (Biologische bundesanstalt, Bundessortenamt und Chemische industrie).

	Cultivar	Harvest Date	Calcium Application	Application Date	Phenological Stage (BBCH)	Fruit Diameter (mm)
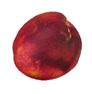	Nectarine ‘*Esmeralda*’	09/05/2023 172 DAFB	Early	06/19/2023 94 DAFB	73	40
			Mid	07/19/2023 124 DAFB	75	48
			Late	08/30/2023 166 DAFB	78	61
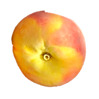	Peach ‘*Tardibelle*’	09/13/2023 175 DAFB	Early	06/19/2023 88 DAFB	73	42
			Mid	07/19/2023 119 DAFB	75	48
			Late	08/30/2023 161 DAFB	78	66

## Data Availability

The raw data supporting the conclusions of this article will be made available by the authors on request.
